# Balancing speed and precision in protein folding: a comparison of AlphaFold2, ESMFold, and OmegaFold

**DOI:** 10.3389/fgene.2025.1715037

**Published:** 2026-01-14

**Authors:** Anna Hýsková, Eva Maršálková, Petr Šimeček

**Affiliations:** 1 Faculty of Science, Central European Institute of Technology (CEITEC), Masaryk University, Brno, Czechia; 2 Faculty of Science, National Centre for Biomolecular Research (NCBR), Masaryk University, Brno, Czechia

**Keywords:** AlphaFold2, ESMFold, foundation models, LightGBM, OmegaFold, protein structure prediction, protein folding, structural bioinformatics

## Abstract

The rapid development of protein structure prediction tools has created a need for systematic performance comparisons to guide method selection for different applications, particularly given the trade-offs between computational speed and prediction accuracy. We benchmarked AlphaFold2, ESMFold, and OmegaFold using 1,337 protein chains deposited in the Protein Data Bank between July 2022 and July 2024, ensuring no overlap with training data, and evaluated predictions using Root Mean Square Deviation (RMSD), Template Modeling score (TM-score), Global Distance Test–Total Score (GDT-TS) and predicted Local Distance Difference Test (pLDDT) metrics. AlphaFold2 achieved the highest median TM-score (0.96), highest median GDT-TS (94%), and lowest median RMSD (1.30 Å), outperforming ESMFold (TM-score 0.95, GDT-TS 90%, RMSD 1.74 Å) and OmegaFold (TM-score 0.93, GDT-TS 89%, RMSD 1.98 Å), with all tools showing reduced accuracy for proteins lacking family annotations, leucine-rich repeats, and NMR-determined structures, while alignment-free methods unexpectedly excelled at *de novo* designed proteins. The performance differences between methods were negligible for many proteins, suggesting that faster alignment-free predictors (10–30 times faster) can be sufficient for numerous applications; we developed LightGBM classifiers using ProtBert embeddings and confidence scores that accurately predict when AlphaFold2’s computational investment is warranted, providing practitioners with actionable guidance for selecting between speed and precision in structural pipelines.

## 1 Introduction

All living organisms—from simple bacteria and algae to plants, fungi, animals, and humans—contain a multitude of proteins that participate in virtually every cellular process (Alberts, 2017; Cooper, 2000). These molecular machines must fold into specific three-dimensional structures, organized hierarchically at four distinct levels: from the linear sequence of amino acids (primary structure), through local folding patterns of α -helices and β -sheets (secondary structure), to the complete three-dimensional arrangement of these elements (tertiary structure), and finally to the assembly of multiple chains into functional complexes (quaternary structure). While the amino acid sequence alone determines the final structure, protein misfolding often leads to disease (Selkoe, 2003). Experimental structure determination through X-ray crystallography, cryo-EM, or NMR spectroscopy remains the gold standard (Smyth and Martin, 2000; Milne et al., 2013; Hu et al., 2021), but these methods are time-consuming, expensive, and not always feasible. This creates an urgent need for reliable computational prediction methods, particularly as the gap between known protein sequences and solved structures continues to widen—with over 254 million sequences known (UniProtKB) but only about 230,444 experimentally determined structures available in the Protein Data Bank (as in January 2025).

The field of protein structure prediction has been transformed by artificial intelligence approaches. The introduction of AlphaFold2 in 2020 marked a watershed moment, achieving near-experimental accuracy (Jumper et al., 2021). This success has spurred the development of alternative approaches, particularly language model-based predictors like ESMFold and OmegaFold that can generate predictions without requiring multiple sequence alignments (Lin et al., 2023; Wu et al., 2022). These newer methods promise faster predictions and potentially better performance on challenging targets like designed or rapidly evolving proteins.

Despite these advances, the field lacks a comprehensive comparison of these tools’ performance on truly novel proteins—structures solved after the tools’ training cutoff dates (Kovalevskiy et al., 2024). Such evaluation is crucial for understanding each method’s strengths and limitations, particularly as these tools become increasingly integrated into structural biology workflows. While the Critical Assessment of Structure Prediction (CASP) (Moult et al., 1995) and Continuous Automated Model EvaluatiOn (CAMEO) (Robin et al., 2021) provide valuable benchmarks, they are limited to participating methods and may not reflect real-world usage patterns.

Here, we present a systematic comparison of AlphaFold2, ESMFold, and OmegaFold using a dataset of over 1,300 protein structures deposited in the PDB between 2022 and 2024. Using multiple evaluation metrics including RMSD (Kufareva and Abagyan, 2012), TM-score (Zhang and Skolnick, 2004), GDT-TS (Zemla, 2003), and pLDDT (Tunyasuvunakool et al., 2021), we assess both overall performance and specific challenging cases. Our analysis reveals that while AlphaFold2 achieves the highest average accuracy, ESMFold and OmegaFold excel in particular niches, especially for proteins with limited homology information. Given 10–30-fold speed difference between alignment-free methods and AlphaFold2, our findings help researchers assess when the faster tools may provide sufficient accuracy for large-scale structural analyses.

## 2 Materials and methods

### 2.1 Dataset

We compiled a benchmark dataset of 1,337 protein structures deposited in the Protein Data Bank (PDB) between July 2022 and July 2024. This temporal restriction ensures no overlap with training data used by AlphaFold2 (cutoff April 2020), ESMFold (June 2020), or OmegaFold (2021). The dataset contains three distinct groups: (1) single-chain monomers (980 structures), (2) small multi-chain complexes (245 structures with 2–6 chains), and (3) *de novo* designed proteins whose sequence does not naturally occur in any living organism (102 structures). *De novo* proteins were identified through PDB annotations marking them as “designed” or “synthetic construct” in the source organism field.

Structures were selected using the RCSB PDB Search API (Rose et al., 2021; Bittrich et al., 2023) with the following criteria: (i) deposition date between July 2022 and July 2024, (ii) protein-only structures without nucleic acids or oligosaccharides, (iii) chain lengths between 20 and 400 amino acids to ensure compatibility with all prediction tools, and (iv) availability of structural information in PDB format. To ensure diversity, structures within monomer and *de novo* protein groups were filtered to have at most 70% pairwise sequence identity.

We developed a custom PDB file parsing pipeline to extract complete amino acid sequences and experimental ${\text{C}}_{\alpha }$ coordinates. The pipeline addresses common challenges in PDB files, including non-standard residue numbering, insertion codes, and post-translational modifications. For modified residues, we reconstructed the original amino acid sequence using BioPython’s extended residue dictionary and MODRES records. Structures containing non-standard residues without clear mapping to canonical amino acids (26 cases) were excluded from the analysis. For NMR-determined structures, which are deposited as ensembles of conformers, we used the first model in the PDB file as the reference structure for all accuracy calculations.

Each structure was annotated with protein family classifications using UniProt and PDBe APIs to map PDB identifiers to Pfam and InterPro database entries. These annotations enable analysis of prediction tools’ performance across different protein families and structural motifs. The numbers of protein structures the dataset contained in various stages of the experiment are stated in Supplementary Table S1. The final curated dataset, including all protein sequences, is available at HuggingFace Hub repository.

### 2.2 Structure prediction tools

Three tools were selected for protein structure prediction: AlphaFold2, ESMFold, and OmegaFold. While alignment-based AlphaFold2 is an obvious choice, considering how widely used it is (Kovalevskiy et al., 2024), language model-based ESMFold and OmegaFold were chosen because they provide promising results with much lower requirements on time and computational power, making them more suitable for large-scale applications (Lin et al., 2023; Wu et al., 2022).

#### 2.2.1 AlphaFold2

We used AlphaFold v2.1.1 running on the institute’s infrastructure with its monomer model and reduced database settings to optimize computational resources. The model architecture consists of two main components: (i) an Evoformer module, which processes multiple sequence alignments (MSAs) and pairwise representations through 48 transformer blocks, and (ii) a structure module that converts the refined representations into 3D coordinates through 8 equivariant transformer blocks with Invariant Point Attention. MSAs were generated using Uniref90, BFD, and MGnify databases. For each sequence, five model predictions were generated and ranked by predicted confidence, with the highest-confidence model (ranked_0.pdb) selected for evaluation.

#### 2.2.2 ESMFold

Predictions were obtained via REST API calls to the ESM Metagenomic Atlas. ESMFold combines two components: (i) the ESM-2 protein language model with 15B parameters, pre-trained on masked sequence prediction, and (ii) a folding head consisting of 48 folding blocks that process sequence and pairwise representations. Unlike AlphaFold2, ESMFold predicts structures directly from single sequences without requiring MSA generation.

#### 2.2.3 OmegaFold

Predictions were performed using OmegaFold v1.0 running on university computational cluster with NVIDIA A40 GPU. OmegaFold employs: (i) OmegaPLM, a 670M parameter language model trained on masked protein sequences, and (ii) a Geoformer architecture that refines the language model representations to be geometrically consistent before structure prediction. Like ESMFold, OmegaFold operates on single sequences without MSA requirements.

All predictions were made for individual protein chains, as both ESMFold and OmegaFold do not support prediction of protein complexes. While AlphaFold2 offers a multimer model, we used its monomer model to ensure fair comparison. The original dataset together with prediction outputs is available at HuggingFace Hub repository.

### 2.3 Evaluation metrics

We employed four complementary metrics to assess prediction quality: RMSD, measuring atomic distance deviation; TM-score, evaluating topological similarity; GDT-TS, quantifying the fraction of residues within distance thresholds after superposition; and pLDDT, reflecting model confidence.

#### 2.3.1 Root mean square deviation (RMSD)

RMSD (1) quantifies the average distance between corresponding ${\text{C}}_{\alpha }$ atoms in superimposed structures:

$$\text{RMSD}=\sqrt{\frac{1}{n}\overset{n}{\underset{i=1}{\sum }}\delta_{i}^{2}}$$ (1)

where n is the number of aligned ${\text{C}}_{\alpha }$ atom pairs and $\delta_{i}$ is the distance between atoms in the i -th pair. To compute RMSD, we first extract ${\text{C}}_{\alpha }$ coordinates from both experimental and predicted structures, then determine the optimal superposition using the Bio.SVDSuperimposer module from BioPython (Cock et al., 2009), which finds the rotation and translation matrices minimizing the RMSD value. While RMSD is widely used, it is sensitive to protein size and can be disproportionately affected by local structural deviations.

#### 2.3.2 Template modeling score (TM-score)

TM-score (2) (Zhang and Skolnick, 2004) evaluates the topological similarity of protein structures while accounting for protein length:

$$\text{TM}-\text{score}=\max\left[\frac{1}{L_{N}}\overset{L_{T}}{\underset{i=1}{\sum }}\frac{1}{1+{\left(\frac{d_{i}}{d_{0}}\right)}^{2}}\right]$$ (2)

where $L_{N}$ is the length of the reference structure, $L_{T}$ is the number of aligned residues, $d_{i}$ is the distance between the i -th pair of aligned residues after superposition, and $d_{0}=1.24\sqrt[{3}]{L_{N}-15}-1.8$ is a length-dependent scaling factor. TM-score ranges from 0 to 1, with values above 0.5 indicating proteins share the same fold and 1 representing perfect structural alignment. Unlike RMSD, TM-score is length-normalized and less sensitive to local structural variations.

#### 2.3.3 Global distance test–total score (GDT-TS)

The Global Distance Test–Total Score (GDT-TS) is a widely used metric in CASP for assessing the similarity between predicted and experimental protein structures, (Zemla, 2003). Unlike RMSD, which is sensitive to outliers, GDT-TS (3) focuses on the fraction of residues that fall within a set of distance thresholds, providing a more robust measure of overall structural agreement.

$$\text{GDT}-\text{TS}=\frac{1}{4}\left(\frac{N_{1\text{Å}}}{L}+\frac{N_{2\text{Å}}}{L}+\frac{N_{4\text{Å}}}{L}+\frac{N_{8\text{Å}}}{L}\right)\cdot 100\%$$ (3)

Here, L is the length of the reference structure, and $N_{x\text{Å}}$ is the number of aligned ${\text{C}}_{\alpha }$ positions whose distances after optimal superposition fall within x Ångström (1, 2, 4, or 8 Å). Each threshold contributes equally to the final score, which ranges from 0 to 100% , with higher values indicating better agreement.

#### 2.3.4 Predicted LDDT (pLDDT)

The predicted local distance difference test (pLDDT) is a confidence metric provided by each prediction tool. For each residue, it estimates the expected agreement between predicted and experimental structures on 0–100 scale. Scores above 90 indicate high prediction confidence. Scores above 70 suggest at least reliable backbone prediction.

For our analysis, we used the mean pLDDT across all residues in each protein chain. While pLDDT correlates with prediction accuracy, high confidence scores do not guarantee correct structure prediction, particularly for challenging targets like intrinsically disordered regions or proteins with limited homology information.

### 2.4 Statistical analysis and annotation

We compared these metrics across our dataset using Kruskal–Wallis tests followed by Dunn’s method with Bonferroni correction for multiple comparisons. The correlation between metrics was assessed using Spearman’s rank correlation coefficient.

Protein chains were mapped to functional annotations using UniProt and PDBe APIs. For family-specific analysis, we focused on Pfam and InterPro families with at least 10 member proteins in our dataset. The experimental method of structure determination (X-ray crystallography, cryo-EM, or NMR) was recorded for each chain to assess potential biases in prediction accuracy.

Predictions were classified as “poor” if they met any of the following criteria: average pLDDT <70 , TM-score <0.5 , RMSD >9 Å, or GDT-TS <50% . The 9 Å RMSD threshold was chosen to match the resolution cutoff used in training AlphaFold2. Statistical significance of family-specific enrichment in poor predictions was assessed using Fisher’s exact test with Benjamini–Hochberg correction for multiple comparisons.

### 2.5 Implementation and availability

All preprocessing was implemented in Python using BioPython (Cock et al., 2009) for structure manipulation and tmtools for TM-score calculation (Xu and Zhang, 2010). Statistical analysis and visualization were performed in R (R Core Team, 2020). The complete dataset, including protein sequences, experimental structures, predictions, and evaluation results is available at HuggingFace Hub, https://huggingface.co/datasets/hyskova-anna/proteins. Source code and documentation are provided at GitHub, https://github.com/ML-Bioinfo-CEITEC/CAoPSPT.

## 3 Results

**FIGURE 1:**
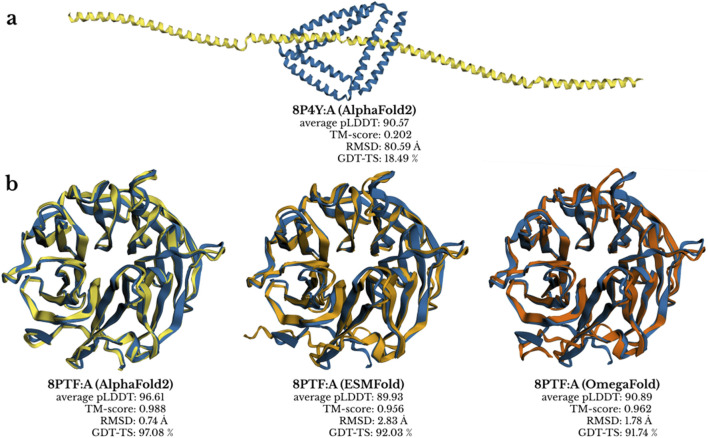
Examples of structure predictions from AlphaFold2 (red), ESMFold (blue) and OmegaFold (yellow) aligned with corresponding experimentally determined structures (green). **(a)** An example of a poorly predicted structure (8P4Y:A) by AlphaFold2. **(b)** Structure of protein 8PTF:A showing varying prediction quality across tools.

Structure predictions were attempted for 1,337 protein chains using AlphaFold2, ESMFold, and OmegaFold. During the initial run, our AlphaFold2 pipeline failed to generate a prediction for one chain (8B2M:A). A subsequent rerun of the pipeline successfully produced a prediction for this chain, indicating that the original failure was due to a transient issue in our university computing service rather than a problem with the structure itself. All chains were successfully predicted by all three tools and form the basis of our evaluation. Selected examples of predictions aligned with their experimental structures are shown in Figure 1.

### 3.1 Comparative performance analysis

All three tools demonstrated generally satisfactory performance, with AlphaFold2 achieving the highest accuracy across all metrics. AlphaFold2 predictions showed the highest median TM-score (0.96), lowest median RMSD (1.30 Å), and highest median GDT-TS (94%), followed by ESMFold (TM-score: 0.95, RMSD: 1.74 Å, GDT-TS: 90%) and OmegaFold (TM-score: 0.93, RMSD: 1.98 Å, GDT-TS: 89%). Consistently, AlphaFold2 displayed the highest confidence in its predictions with median pLDDT of 92.65, compared to 87.40 for ESMFold and 89.00 for OmegaFold (see Supplementary Figure S1). These differences were statistically significant across tools for all four metrics (Kruskal–Wallis test, p<0.001 ); *post hoc* Dunn tests with Bonferroni correction showed significant pairwise differences between all method pairs (p<0.01) .

### 3.2 Metric correlations and their dependencies on sequence length and other factors

We observed significant correlations between prediction confidence (pLDDT) and accuracy metrics. Most notably, there was a negative correlation between average pLDDT and RMSD (Spearman’s ρ=−0.56 , −0.61 , and −0.68 for AlphaFold2, ESMFold, and OmegaFold, respectively), positive correlations between average pLDDT and TM-score ( ρ=0.60 , 0.66, and 0.71), and between average pLDDT and GDT-TS ( ρ=0.55 , 0.60, and 0.67). All reported correlations were statistically significant ( p<0.001 ; see Supplementary Figure S2). The correlations were strongest for ESMFold and OmegaFold, suggesting that their confidence scores more accurately reflect prediction quality than those of AlphaFold2.

While low-confidence predictions rarely achieved good accuracy metrics, we found numerous cases of incorrect structures with high pLDDT scores across all tools (see Supplementary Figure S3).

Analysis of sequence length dependency also revealed interesting patterns. While RMSD showed weak correlation with sequence length, TM-score and GDT-TS displayed stronger positive associations, particularly for AlphaFold2 (TM-score: ρ=0.41 , p<0.001 ). This suggests that predictions for shorter proteins ( <100 amino acids) tend to achieve lower TM-scores across all tools, though this trend is less pronounced in RMSD values due to the metric’s inherent length dependency. ESMFold and OmegaFold showed weaker but still significant correlations with sequence length ( ρ=0.30 and ρ=0.29 , respectively, for TM-score).

The experimental method used for structure determination significantly influenced prediction accuracy (Supplementary Figure S4). All tools performed best on X-ray crystallography structures (median RMSD: 1.24 Å, 1.65 Å, and 1.89 Å for AlphaFold2, ESMFold, and OmegaFold, respectively) but struggled with NMR-determined structures (median RMSD: 2.31 Å, 2.89 Å, and 3.12 Å). This pattern likely reflects both the inherent flexibility of proteins amenable to NMR analysis and the predominance of X-ray structures in training data.

**FIGURE 2:**
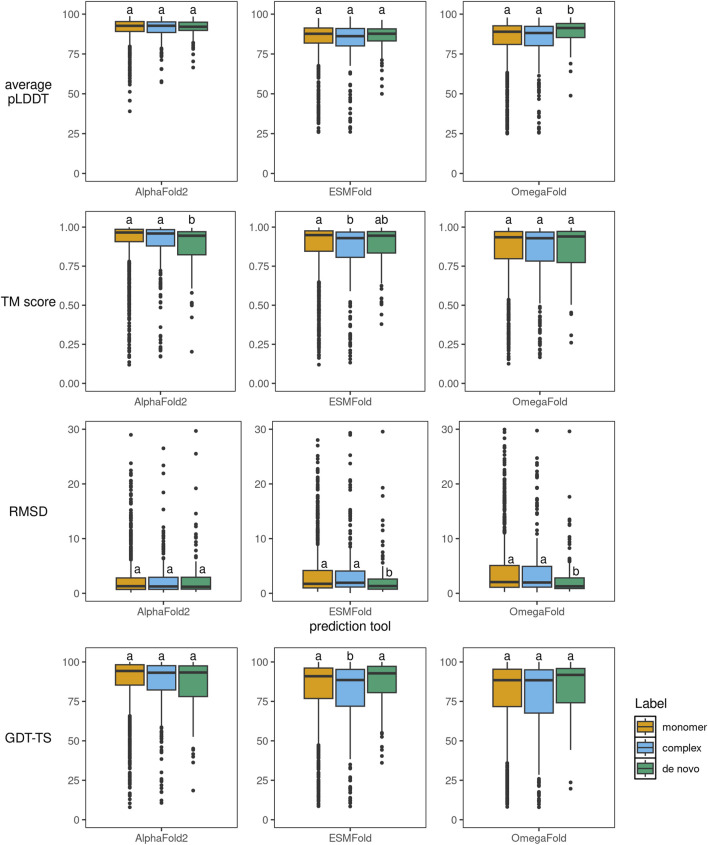
Dependency of average pLDDT, TM-score, RMSD, and GDT-TS on the type of protein chain being predicted. The differences between groups were tested by Kruskal–Wallis test, *post hoc* comparisons were done using Dunn’s method with a Bonferroni correction for multiple tests. Different letters above boxplots indicate statistically significant differences among label groups within each prediction tool (compact letter display; groups sharing a letter are not significantly different). Sample points with RMSD greater than 30 Å are omitted from the visualization for better clarity.

When comparing performance across different protein types (monomers, complexes, and *de novo* proteins), we observed an interesting pattern. While all tools generally performed similarly across these categories, there are two notable exceptions. First, ESMFold and OmegaFold achieved significantly lower RMSD values for *de novo* proteins compared to natural proteins. Statistical significance was assessed using Kruskal–Wallis tests with Dunn *post hoc* comparisons and Bonferroni correction (Figure 2). Second, AlphaFold2 showed a unique weakness with *de novo* proteins, achieving significantly lower TM-scores for these proteins compared to monomers and complexes. This suggests that language model-based tools may have an advantage in predicting structures of artificial proteins where evolutionary information is limited.

### 3.3 Analysis of prediction failures

**FIGURE 3:**
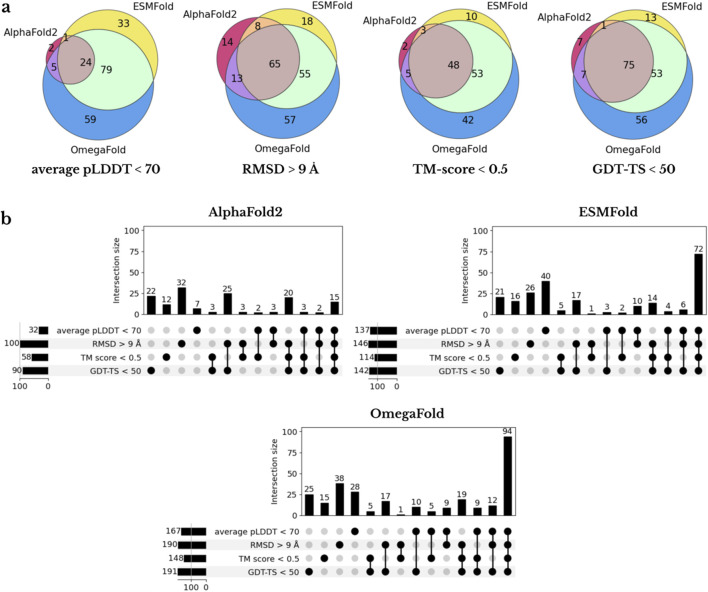
Comparison of the overlap of poorly predicted protein chains. **(a)** Venn diagrams show the overlap of poorly predicted chains among the three structure prediction tools (AlphaFold2, ESMFold, and OmegaFold) for each evaluation metric: average pLDDT <70 , TM-score <0.5 , RMSD >9 Å, and GDT-TS <50% . **(b)** UpSet plots visualize the intra-tool failure patterns by quantifying how many predictions fail each individual metric and every combination of metrics.

We classified predictions as incorrect if they met any of the following criteria: average pLDDT <70 , TM-score <0.5 , RMSD>9 Å, or GDT-TS <50% . AlphaFold2 produced the fewest incorrect predictions (8.9% of total), followed by ESMFold (13.0%) and OmegaFold (16.8%). The overlap of prediction failures between tools was limited, suggesting complementary strengths (Figure 3).

Analysis of protein families revealed that proteins lacking Pfam annotations were particularly challenging for AlphaFold2 but not for ESMFold or OmegaFold, highlighting the importance of evolutionary information in AlphaFold2’s predictions. Conversely, viral proteins, especially from coronavirus, were better predicted by AlphaFold2 than by the language model-based tools. All tools showed reduced accuracy for proteins containing leucine-rich repeats or von Willebrand factor A-like domains, suggesting these structural motifs pose particular challenges for current prediction methods.

The analysis of protein family associations revealed distinctive patterns in prediction accuracy. Notably, AlphaFold2 showed significantly reduced performance for proteins lacking Pfam family annotations (odds ratio = 0.67, p<0.01 ), while ESMFold and OmegaFold maintained consistent performance regardless of family assignments. This pattern was also observed with InterPro annotations, highlighting AlphaFold2’s dependence on evolutionary information.

Certain protein families were consistently well-predicted across all tools. These included protein kinase domains (PF00069, IPR000719), the SH2 domain (IPR000980), and the NAD(P)-binding domain superfamily (IPR036291). Conversely, all tools struggled with leucine-rich repeats (IPR001611, IPR003591) and von Willebrand factor A-like domains (IPR036465), suggesting these structural motifs remain challenging for current prediction methods.

Interestingly, several protein families showed tool-specific prediction patterns. AlphaFold2 excelled at predicting viral protein families, particularly the viral RNA-dependent RNA polymerase (PF00680, IPR001205) and coronavirus-specific proteins (PF05409, IPR043503), achieving significantly better accuracy than ESMFold or OmegaFold (p<0.001) . Conversely, the S-adenosyl-L-methionine-dependent methyltransferase superfamily (IPR029063) showed markedly different prediction quality between AlphaFold2 (odds ratio = 1.49) and the language model-based tools (odds ratio = 0.31 and 0.25 for ESMFold and OmegaFold respectively, p<0.01 ).

### 3.4 Prediction of structure determination success using machine learning

To assess and anticipate potential failures in structure prediction, we trained gradient boosting LightGBM models (Ke et al., 2017) separately for AlphaFold2, ESMFold, and OmegaFold. For each method, models were trained both *with* and *without* inclusion of the model-specific confidence estimate (pLDDT), resulting in six model configurations in total. Input features included ProtBert BFD sequence embeddings (Brandes et al., 2022), sequence length, experimental acquisition method, and pLDDT where applicable.

Model performance was evaluated using mean squared error (MSE) and coefficient of determination $\left(R^{2}\right)$ . In addition, regression outputs were thresholded at TM-score <0.8 to assess the ability to identify low-quality predictions, reporting ROC-AUC and F1-score (Supplementary Table S2). Across all three structure prediction methods, the models achieved strong regression performance ( $R^{2}\approx 0.53$ –0.76) and reliably discriminated low-quality structures (ROC-AUC ≈0.88 –0.95). While inclusion of pLDDT consistently improved performance, models trained without this feature retained substantial predictive power, indicating robustness to potential confidence-related bias.

**FIGURE 4:**
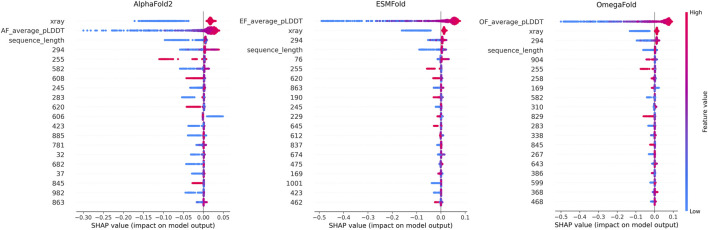
SHAP analysis of LightGBM models predicting TM-score. SHAP summary plots for LightGBM regressors trained separately for AlphaFold2, ESMFold, and OmegaFold using ProtBert sequence embeddings, sequence length, experimental acquisition method, and model-specific confidence estimates (pLDDT) as input features. Features are ordered by mean absolute SHAP value, indicating their overall influence on predicted TM-score. Each point represents an individual protein chain, colored by feature contribution.

Feature contributions were interpreted using SHAP analysis (Figure 4). Across all methods, higher pLDDT values contributed positively to predicted TM-scores, whereas shorter sequence length and experimental acquisition methods other than X-ray crystallography showed negative contributions. Selected embedding dimensions also showed consistent contributions, reflecting sequence-level patterns associated with prediction difficulty.

## 4 Discussion

Since the beginning of this decade, structural biology and protein structure prediction fields have undergone a significant transition. Currently, there are two large projects dealing with this issue: CASP (Moult et al., 1995) and CAMEO (Robin et al., 2021). While AlphaFold2 has participated in both CASP14 and CAMEO, ESMFold has entered only CASP15, and OmegaFold has not been included in either. However, both ESMFold and OmegaFold have been subsequently evaluated on CAMEO and CASP15 datasets by independent research groups (Moussad et al., 2023; Huang et al., 2023). There are also a few publications dealing with the comparison of protein structure prediction tools, but they usually focus mainly on AlphaFold2 and similar tools (e.g., ColabFold) (Kalogeropoulos et al., 2024) or perform the evaluation on a particular set of proteins, namely, human proteins (Manfredi et al., 2024; Manfredi et al., 2025), snake venom toxins (Kalogeropoulos et al., 2024), and nanobodies (Valdés-Tresanco et al., 2023). This paper tries to increase our understanding by creating an inclusive dataset of protein structures recently added to PDB.

The key finding of this work is that AlphaFold2 outperforms ESMFold and OmegaFold on a majority of proteins in the dataset, measured by both RMSD, TM-score, and GDT-TS. When comparing the two protein language-based models, ESMFold seems to be a slightly better choice, as it produced fewer incorrect structures than OmegaFold and achieved significantly better median RMSD and TM-score. Still, the difference in performance between ESMFold and OmegaFold is much smaller compared to the gap between both of these tools and AlphaFold2.

While all three tools rarely produce a good prediction with low confidence, wrong structures with a high average pLDDT are outputted quite frequently. Our analysis revealed that prediction accuracy is influenced by various factors. All three tools performed best when predicting proteins whose experimental structure was determined by X-ray crystallography, while structures determined by NMR proved to be the most challenging. Because NMR is typically used to determine the structures of small proteins, a corresponding decrease in prediction accuracy is observed for shorter sequences. Additionally, for NMR-determined structures, evaluation against a single representative conformer from an ensemble may further contribute to the observed reduction in apparent prediction accuracy.

Interestingly, proteins without family annotations proved particularly difficult for AlphaFold2 but did not change the performance of ESMFold and OmegaFold. A possible explanation is that proteins belonging to no family lack homologs with a known structure, which AlphaFold2 could use as a template during the prediction. In contrast, ESMFold and OmegaFold do not rely on MSAs and modeling templates, so their performance remained largely unaffected.

Our analysis shows several key insights, yet certain constraints of our study must be noted. First, the dataset does not contain only proteins whose experimental structure was previously unknown but also proteins that were just recently analyzed again, usually in different conditions. This might be an advantage for AlphaFold2, which uses a reduced PDB database for template searching during the prediction process. Moreover, the whole analysis focuses only on single protein chains without the context of their interacting partners, which might be crucial for structure formation, especially in protein complexes. Additionally, speed comparisons should be interpreted with caution, as pipelines for OmegaFold and AlphaFold2 predictions with different hardware configurations, potentially affecting relative performance metrics. Last but not least, all the protein chains in the dataset have a maximum length of 400 amino acids due to using ESMAtlas API.

The performance patterns we observed reflect fundamental architectural differences between these approaches. AlphaFold2’s superior accuracy stems from leveraging evolutionary information through MSAs, but this becomes a limitation for *de novo* proteins where we observed reduced TM-scores. In contrast, language models learn protein grammar from sequence patterns alone, potentially capturing more general folding principles. The limited overlap in prediction failures between tools suggests complementary error modes that could be exploited through ensemble approaches, though computational costs may be prohibitive for large-scale applications.

The recent proliferation of AlphaFold3 (Abramson et al., 2024; Callaway, 2024) and its alternatives, including Chai-1 (Chai Discovery, 2024), Boltz-1 (Wohlwend et al., 2024), and HelixFold3 (Liu et al., 2024), demonstrates the community’s commitment to structure prediction. Independent benchmarks have begun evaluating these tools: FoldBench (Xu et al., 2025), evaluating 1,522 biological assemblies across nine tasks, found AlphaFold3 consistently outperforming alternatives across most categories, though all methods showed concerning failure rates exceeding 50% for antibody-antigen predictions. For protein-peptide interactions, newer models achieve dramatic improvements, with success rates of 70%–80% under stringent criteria compared to 53% for AlphaFold2-multimer, and Protenix reaching 80.8% accuracy (Zhou et al., 2025). However, as shown in (Škrinjar et al., 2025), protein-ligand predictions reveal a critical limitation: current methods largely memorize poses from training data rather than genuinely predicting novel interactions, particularly struggling with ligands not seen in their training sets. Practical deployment is being facilitated by tools like ABCFold (Elliott et al., 2025), which standardizes inputs and outputs across different methods. This proliferation of capable yet specialized tools, each with distinct strengths and limitations, reinforces our findings: optimal structure prediction requires matching tools to specific tasks based on target type, available computational resources, and accuracy requirements rather than relying on any single universal solution.

## Data Availability

The datasets generated/analyzed for this study can be found in the HuggingFace Hub repository at https://huggingface.co/datasets/hyskova-anna/proteins. The source code and documentation are provided on GitHub: https://github.com/ML-Bioinfo-CEITEC/CAoPSPT.
