# Supplementary material for: Balancing speed and precision in protein folding: a comparison of AlphaFold2, ESMFold, and OmegaFold
Source: Front Genet. 2026 Jan 14;16:1715037. doi: 10.3389/fgene.2025.1715037 (PMC12844563; doi:10.3389/fgene.2025.1715037)
Supplement: Supplementary file 1 [file DataSheet1.pdf]

Supplementary Material

1 SUPPLEMENTARY TABLES

Table S1. Size of the dataset in various stages of the experiment. (A) Number of hits in PDB database. (B) Number of randomly selected structures. (C) Number of structures with PDB files successfully preprocessed. (D) Final number of chains used in the evaluation dataset (successfully predicted by all three tools).

Group	PDB hits (A)	Selected structures (B)	Successfully processed (C)	Evaluated chains (D)
Monomers	3830	1000	980	980
Small Complexes	3988	250	245	255
De Novo Proteins	139	103	102	102
In Total	7957	1353	1327	1337

Table S2. Performance of LightGBM regressors predicting TM-score for AlphaFold2, ESMFold, and OmegaFold. Models were trained with and without pLDDT. Regression performance is reported using mean squared error (MSE) and coefficient of determination (R^2). To quantify low-quality detection, regression predictions were thresholded at $TM - score < 0.8$ and evaluated using ROC-AUC and F1-score.

Method	pLDDT	MSE	R^2	ROC-AUC	F1
AlphaFold2	Yes	0.011	0.616	0.883	0.744
AlphaFold2	No	0.013	0.528	0.886	0.672
ESMFold	Yes	0.01	0.719	0.946	0.765
ESMFold	No	0.015	0.606	0.932	0.759
OmegaFold	Yes	0.011	0.764	0.934	0.794
OmegaFold	No	0.018	0.631	0.918	0.739

2 SUPPLEMENTARY FIGURES

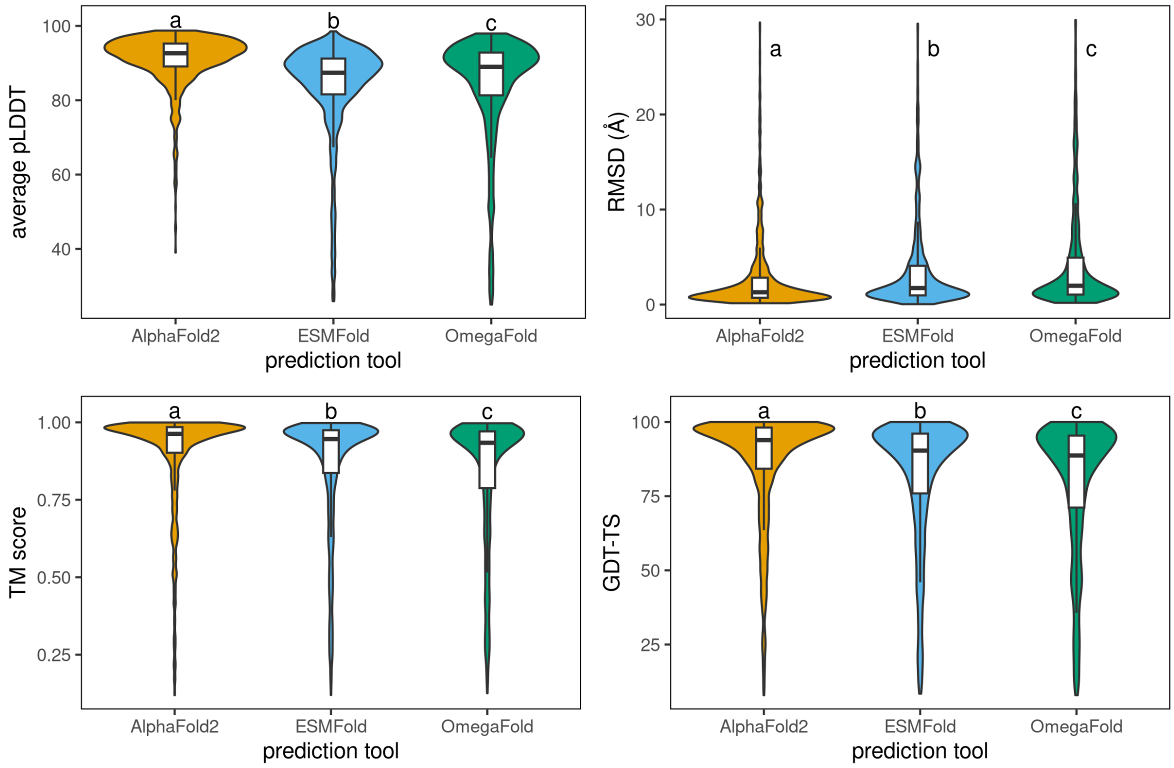

Figure S1. Performance comparison across prediction tools. Distribution of RMSD values, TM-scores, pLDDT, and GDT-TS. Box plots show median, quartiles, and outliers. All pair comparisons have been statistically significant ($p < 0.05$).

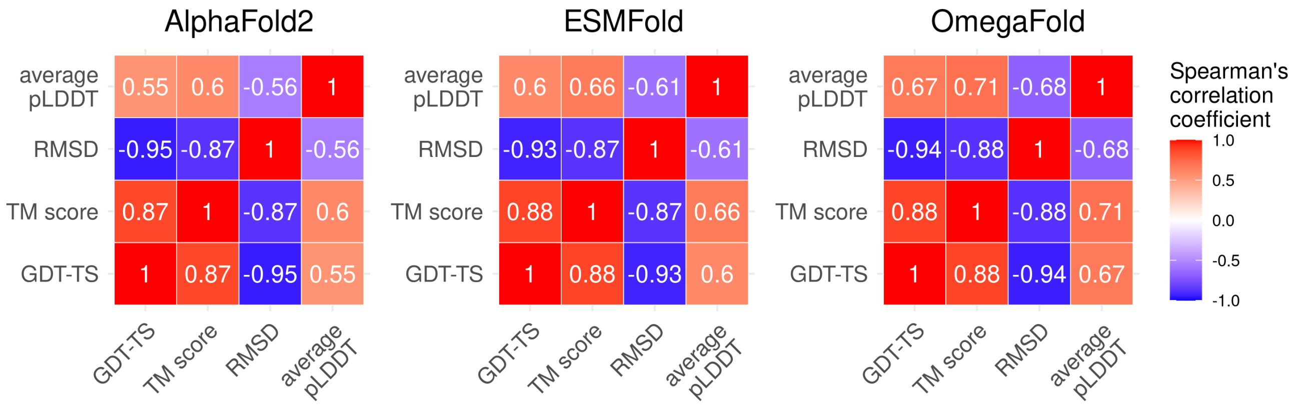

Figure S2. Correlation analysis between prediction metrics. Heatmaps show Spearman's correlation coefficients between average pLDDT, RMSD, TM-score, and GDT-TS for each prediction tool. All correlations are statistically significant ($p < 0.001$).

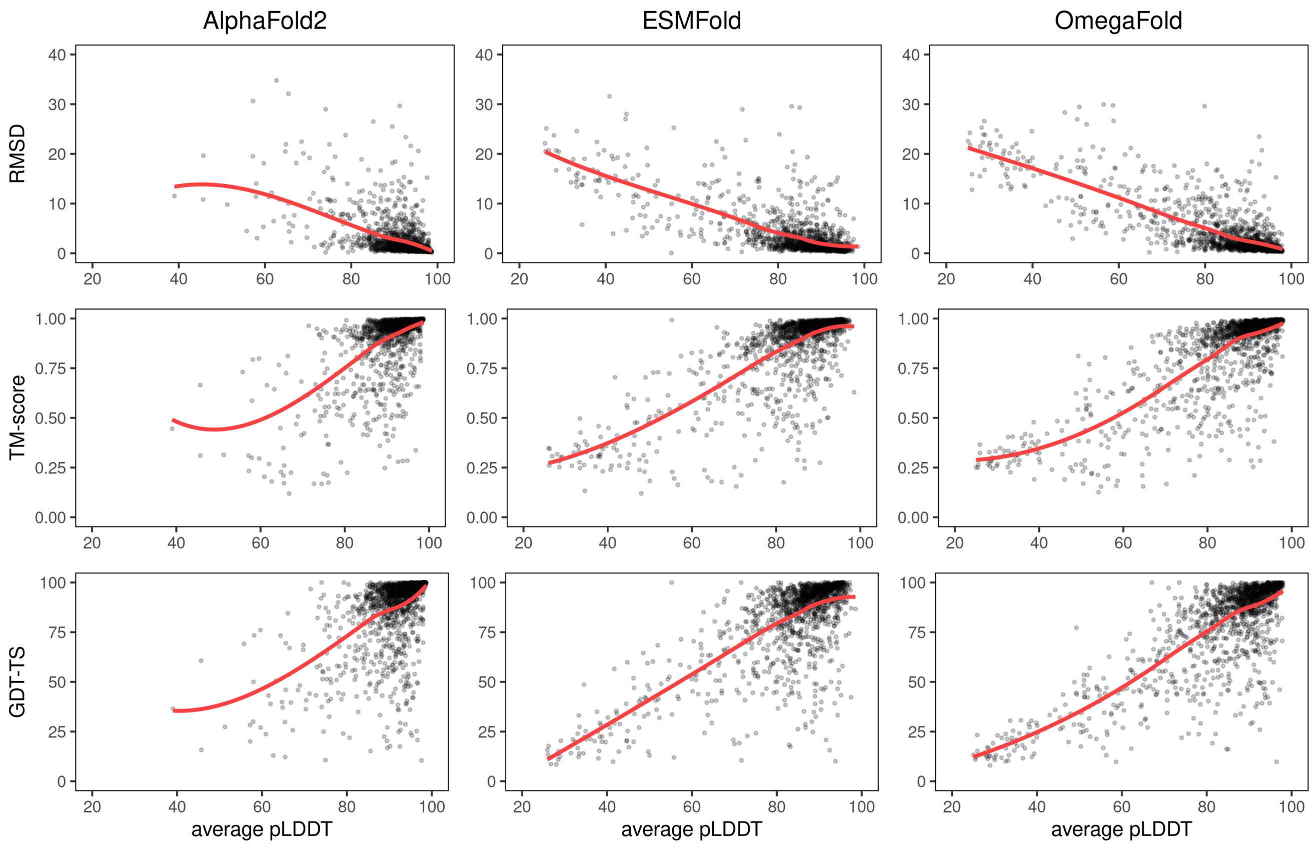

Figure S3. Dependency of RMSD, TM-score and GDT-TS on average pLDDT of structures generated by different tools. The LOESS curve (red) was obtained by locally estimated scatterplot smoothing. Sample points with RMSD greater than 40 Å are omitted from the visualization for better clarity.

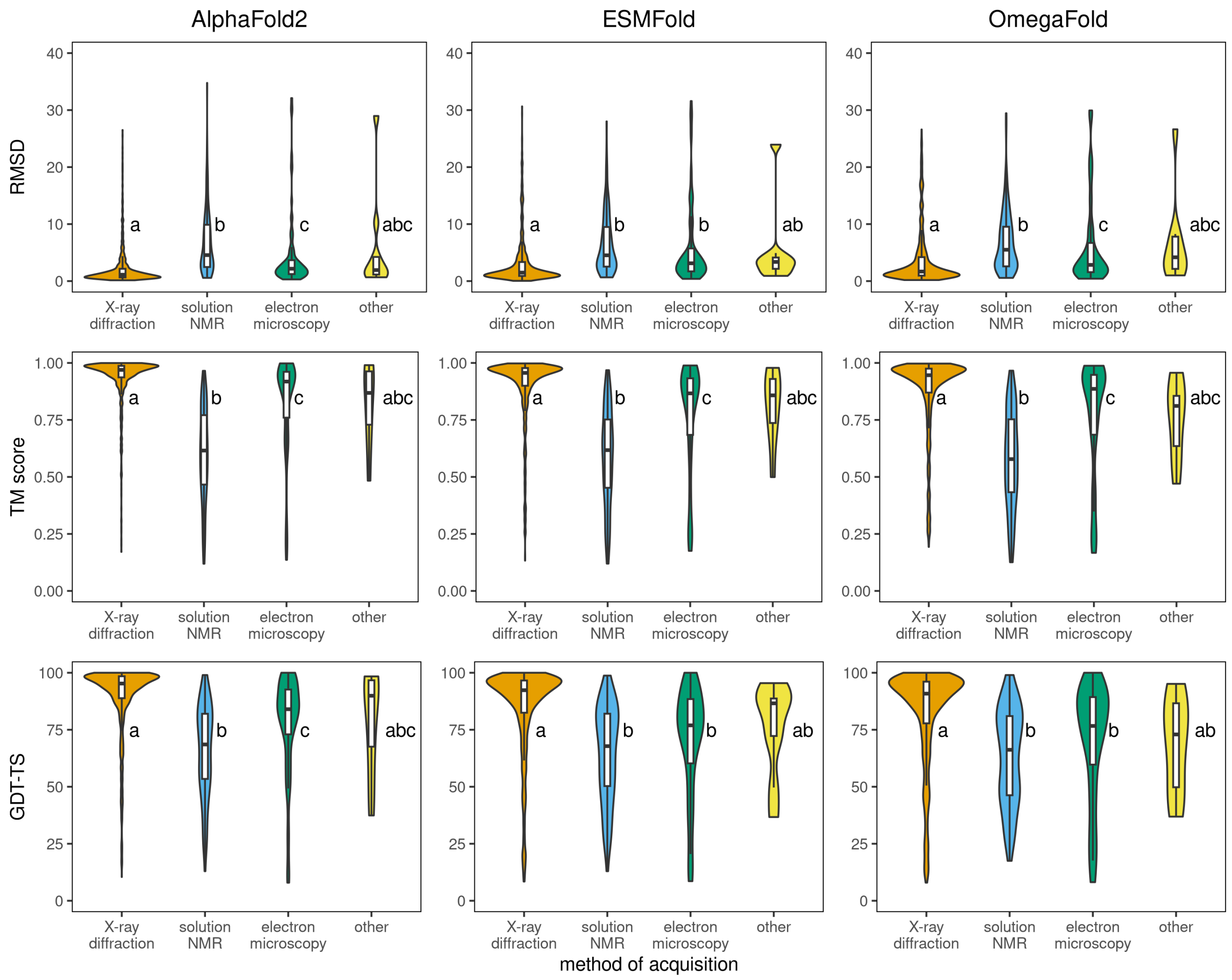

Figure S4. Dependency of RMSD, TM-score and GDT-TS on the experimental method of acquisition of the protein chain structure. The differences between groups were tested by Kruskal-Wallis test, post-hoc comparisons were done using Dunn's method with a Bonferroni correction for multiple tests. Different letters above violins indicate statistically significant differences between acquisition methods (compact letter display; groups sharing a letter are not significantly different). Sample points with RMSD greater than 40 Å are omitted from the visualization for better clarity.

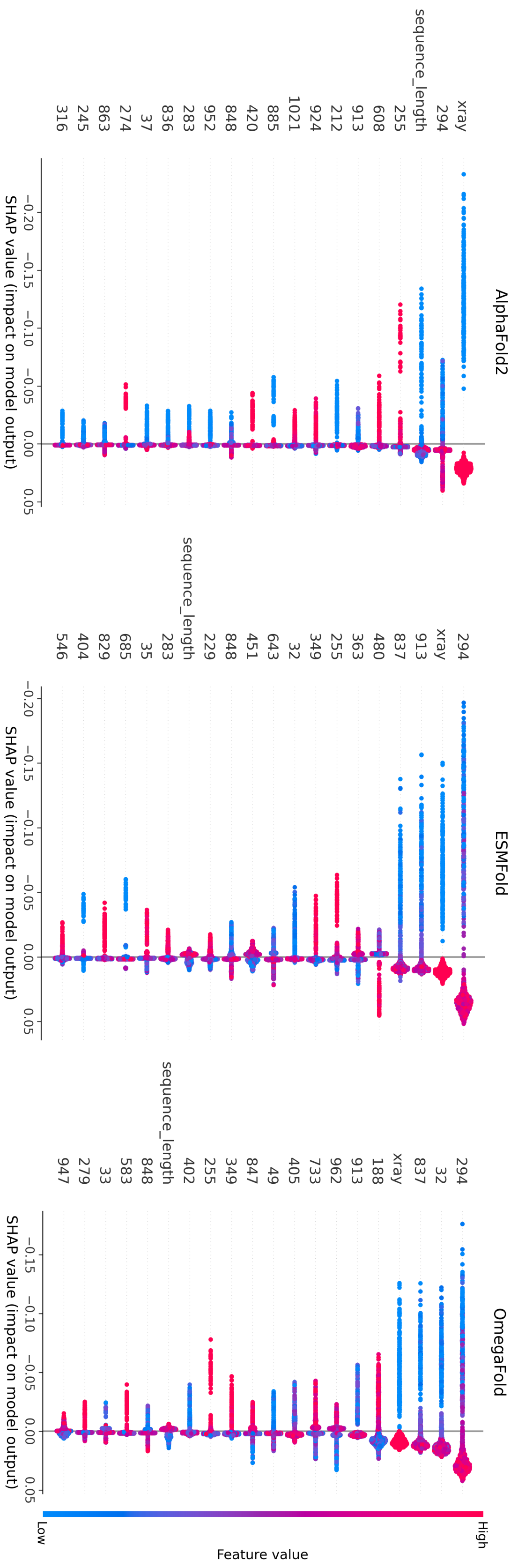

Figure S5. SHAP analysis of LightGBM models predicting TM-score – omitting pLDDT. SHAP summary plots for LightGBM regressors trained separately for AlphaFold2, ESMFold, and OmegaFold using ProtBert sequence embeddings, sequence length, experimental acquisition method, but not model-specific confidence estimates (pLDDT) as input features.
